# Procedural skills training for Canadian medical students participating in international electives

**Published:** 2015-04-20

**Authors:** Joseph Margolick, David Kanters, Brian H Cameron

**Affiliations:** 1 Department of Surgery, Division of General Surgery, University of British Columbia, Vancouver, BC; 2 International Surgery Desk, Department of Surgery, McMaster University, Hamilton, ON

## Abstract

**Background:**

International medical electives (IMEs) are unique learning opportunities; however, trainees can risk patient safety. Returning medical students often express concern about doing procedures beyond their level of training. The Canadian Federation of Medical Students has developed guidelines for pre-departure training (PDT), which do not address procedural skills. The purpose of this research is to determine which procedural skills to include in future PDT.

**Methods:**

Twenty-six medical students who returned from IMEs completed surveys to assess PDT. Using a Likert scale, we compared procedures performed by students before departing on IME to those performed while abroad. We used a similar scale to assess which procedures students feel ought to be included in future PDT.

**Results:**

There was no significant increase in number of procedures performed while on IME. Skills deemed most important to include in future PDT were intravenous line insertion, suturing of lacerations, surgical assisting and post-operative wound care.

**Conclusions:**

Pre-departure training is new and lacks instruction in procedural skills. Over half the students rated several procedural skills such as IV line insertion, suturing, assisting in surgery, post operative wound management and foley catheterization as important assets for future PDT.

## Introduction

Growing interest in global health is creating opportunities for medical students to train abroad.^1^ Participation in international medical electives (IME) has doubled in the past 20 years^1^ with 30% of Canadian medical students now spending some time training overseas.^2^ The benefits of international work during medical school are numerous.^3^,^4^ Mutchnick, Moyer and Stern reviewed 42 publications and described key benefits of IMEs such as professional skill and personal development.^3^ International medical electives are a opportunities to promote cultural competency, communication skills and compassion. Training abroad can break ground for a lifetime of service in the spirit of equitable healthcare delivery and research has shown that trainees who complete IMEs are more likely to volunteer for international work in the future.^5^ However, recent attention has been paid to whether the level of training of first and second year medical students is adequate for electives in low-resource settings.

Many electives are undertaken in resource-poor environments creating unique ethical dilemmas for students. Unprepared and inexperienced trainees can be safety risks to themselves and patients.^4^–^7^ The role of the student on an IME is often location-dependent and can be difficult to predict. For example, some hospitals with minimal resources depend on trainees to practice independently and elective students may find themselves functioning as physicians.^4^–^6^

To mitigate situations of ethical uncertainty, many Canadian medical schools have implemented pre-departure training (PDT) courses. Pre-departure training is relatively new at many medical schools^6^ and despite every medical school in Canada sending students on IEs, until recently there were no national standards for PDT. This led to a large variability in the level of training students had prior to departure. A recent survey of Canadian medical schools showed that only 64% of medical schools have mandatory PDT programs.^8^ These programs varied in content, and ranged in length from 30 minutes to 30 hours.^5^

In response to the variability in PDT, the Canadian Federation of Medical Students (CFMS) developed PDT guidelines in 2012. The guidelines focus on five competencies: personal health, travel safety, cultural awareness, language awareness and ethical considerations.^2^,^6^ The Action Global Health Network produced a similar pre-departure guide for students, which focuses on similar domains.^9^

While the current CFMS guidelines comprehensively focus on ethical and cultural dilemmas, guidelines for training in procedural or surgical skills are absent. While on an IME, students may be asked to perform procedures independently or with little supervision.^4^ This practice engenders greater risk of violating ethical principles.^5^ The amount of training required to ensure competence in a specific skill may vary amongst trainees. However, the practical and theoretical knowledge required to perform procedures at the home institution should not differ when a trainee goes abroad. Competence is defined broadly as the ability to safely care for patients in a knowledgeable manner whilst maintaining acceptable standards of professional behavior.^10^

Many medical students returning from IMEs have expressed concern about doing procedures beyond their scope of training.^7^ Thus, if Canadian medical schools are to continue supporting IMEs, a minimum level of competence in certain basic skills should be required. Without this we are shifting the burden of training to those whose resources are limited. Furthermore, language barriers may impede effective teaching of potentially dangerous procedures. Departing students are often cautioned that participation in procedures should match their current level of training. This is often not the reality and students may feel pressured to perform procedures above their level. Indeed many students return from electives wondering if their participation had been harmful to patients.^6^

The purpose of our research was to inform curriculum development for pre-departure procedural skills training. We wanted to compare medical students’ level of exposure to procedures before their IME to their actual exposure during the electives, assess which skills students think are important to include in future PDT and determine if this varies based on whether the IME is surgical.

## Method

All second year McMaster University medical students who completed an IME in the summer of 2012 were eligible to participate in the study. Students were contacted by email to complete the online survey (LimeSurvey© www.limesurvey.org ) and participant consent was obtained. Each participant was given a unique access code to ensure confidentiality and participants were free to exit the survey at any point. The survey can be found in Appendix A.

Demographic data regarding electives were obtained. Participants then rated their pre-elective exposure to eight basic procedural skills on a five-point Likert scale ranging from “never observed” to “performed without supervision.” Participants used the same scale to rate their exposure to procedures during their international elective.

Participants rated the importance of PDT in 11 procedural skills and surgical concepts using a five-point Likert scale. On the five-point scale a “one” indicated the skill was deemed “not important” for future PDT, a three out of five rated the skill as “optional” for future PDT while a “five” rated it is “very important” to include in future PDT.

Descriptive statistics were computed for patient demographic characteristics and IME details. Fisher’s exact test was used to assess differences in procedural exposure prior to and during IME. Between groups (students who went on surgical IMEs vs non-surgical electives) was assessed using Welch’s t test. Variables determined to have p-value < 0.05 were deemed significant.

## Results

Forty-eight students from the McMaster Class of 2014 who undertook international electives in their second year (23.3% of the class) were invited to participate in the study and 26 (54%) completed the survey. All students who participated in the survey stated they had attended pre-departure training sessions. Only one student reported any PDT in procedural skills. Elective characteristics are summarized in Table 1. Medical students travelled to 16 different countries located in Africa, Asia, The Middle East, South/Central America, Europe and Australia (Table 1). Participants completed electives in several different fields of medicine, the most common being surgery (30%), internal medicine (37%), pediatrics (23%), public health (19%) and emergency medicine (15%; Table 1).

Nineteen percent of participants went on elective to a low-income country defined by The World Bank as having a gross national income (GNI) per capita of 0 to 1,025 USD.^11^ Thirty-one percent of students went on elective to lower-middle income countries defined as a GNI per capita of 1,025 to 4,035 USD.^11^ Four percent of students went to middle-income countries (GNI per capita of 4,035 to 12, 457 USD) and 46% of participants went on elective to high-income countries (GNI per-capita greater than 12,475 USD).^11^ Seventy-three percent of students travelled to countries outside of the United States or Western Europe.

There was no significant difference between the number of procedures performed by medical students on elective and prior to the elective (Table 2). Participants did not insert more or less IVs, intubate, suture, hand tie, insert chest tubes or Foley catheters any more on elective than previously at their home school. Students did observe significantly less drain suturing (p < 0.05) and airway intubations (p < 0.01) while on electives (Table 2).

Participants rated the importance of PDT in a variety of common procedures on a five-point scale, with five indicating training as very important for future PDT. The most important skills to incorporate in future PDT were identified by students as: inserting IV lines (*χ̄* = 4.2/5), suturing lacerations (*χ̄* = 4.1/5), assisting in surgery (*χ̄* = 3.9/5), post-operative wound care (*χ̄* = 3.9/5) and foley catheterization (*χ̄* = 3.8/5; Table 3). Sixty-four percent of participants rated pre-departure training in IV insertion as being either important or very important for future PDT. Seventy percent of participants rated learning to suture a laceration as either an important or very important skill to learn during PDT while 65% of students rated training in surgical assisting an important or very important component of PDT. Sixty-three percent of participants stated that post-operative wound care knowledge should be an important component of future PDT. Results are summarized in Table 2.

Students who went on a surgical IME rated the importance of chest tube insertion training higher and training in drain/tube suturing as more important than those who went on a non-surgical IME (Table 4). There were no other significant differences between students doing surgical electives and those doing non-surgical electives in rating the importance of procedural skills.

## Discussion

There was no increase in the number of medical or surgical procedures performed by medical students while on elective abroad compared to their pre-elective experience. However, well over half the students rated training in IV cannulation, laceration suturing, assisting during surgery and post-operative wound care as important for PDT. Furthermore, all of the procedures except chest tube insertion were rated higher than 3.0/5 in importance for future PDT (Table 2). Our data clearly indicates that medical students would value procedural skills training as an addition to the current PDT.

Research has shown that many health care practitioners at host institutions in low-resource countries assume that medical students from western countries possess more skill than they actually do.^4^,^6^ Elit and colleagues interviewed Canadian medical students returning from IME.^7^ In their study, several students recounted situations in which patients had requested that the student perform a procedure despite there being a more competent local health care worker available. Students had also expressed concern about carrying out procedures with poor supervision and being required to do tasks that exceeded their level of training.^7^ Respondents stated that while on elective, expectations for supervision were often unclear. In contrast, Canadian supervisors are generally aware of students’ level of training and can dictate their involvement in procedures. On an IME, students may be required to use their own judgment.^7^

The benefits of IME are well documented and experiencing health care abroad creates a more robust medical education in global health.^3^,^4^ Additionally, the changing landscape of international healthcare creates a need for global health competency in future physicians.^12^ However, with more and more medical students going away on IMEs, concerns of students doing procedures beyond their capacity should be addressed. Electives abroad are generally unregulated and a wide variation in supervision exists. Indeed most students are placed with a local community through their own volition and investigation rather than through a high-profile, well organized group like Doctors Without Borders.^13^

Given the sheer volume of students going on IME and the variety of locations they visit, more stringent oversight of IME educational experience should be considered. International electives are likely to continue growing in popularity and applying North American professional norms is especially challenging in resource-limited settings.^12^ One approach to dealing with the issue of medical students performing procedures beyond their scope is to increase procedural skills training in PDT in addition to discussion of cultural awareness and ethics. Our research found that a majority of medical students would support this initiative.

It is prudent to attempt to reduce circumstances of moral uncertainty encountered by medical students; working through ethical dilemmas requires the ability to relate past experiences.^14^

The goal of an IME should be mutual and reciprocal benefit between the sending institution and receiving institution. While the student’s objective for IME should focus on learning rather than helping, we argue that spending some extra time in PDT to teach basic procedural skills will improve IME outcomes for both students and hosts. We want to minimize the burden of training for host communities, especially those in resource poor environments. Host communities face the problem of having to spend time, money and resources training students who are unlikely to return as practicing physicians to the exact setting of their international elective.

Limitations of this study include its retrospective design and lack of comparison control groups. The survey was also limited by a small sample size. The study was also restricted to past elective participants from one medical institution (McMaster University) therefore the generalizability of the results is limited. The results are based on students’ self reports and a few students completed most but not all the questionnaire items. Despite these limitations, this is the first paper to our knowledge that identifies specific procedures for skills training in PDT in Canada.

What we propose is to give the medical students teaching and practice in the basic procedural skills they may encounter while on elective. This will make for a more robust learning experience and will reduce some unease of performing procedures for the first time while abroad. Future research is needed to optimize and standardize PDT curriculum in procedural skills.

## Disclaimer

The views expressed in the article are the views of the authors alone and are not an official position of any institution. The authors declare no conflict of interest. No funding was granted for this research

## Figures and Tables

**Table 1:** Elective demographic information

Location of Elective	Proportion of students by destination

Africa	19.2%
Asia	19.2%
South/Central America	19.2%
Middle East	15.2%
Europe	19.2%
North America	4.0%
Australia	4.0%

Length of Elective	Proportion of electives by lengths

< 3 weeks	11.5%
3–6 weeks	80.7%
< 6 weeks	7.8%

Elective Specialty	Number of students

Surgery	30.0%
Internal Medicine	37.0%
Pediatrics	23.0%
Family Medicine	11.5%
Psychiatry	0.0%
Obstetrics	3.8%
Public Health	19.2%
Emergency Medicine	15.4%
Cardiology	7.7%
Other	11.5%

**Table 2:** Difference between procedures performed prior to and during international electives

Procedure		Pre-elective experience (% of respondents) N = 26	Elective experience (% of respondents) N = 26	*p* –value	Odds ratio
Inserting IV lines	Performed with or without supervision	0.16	0.27	0.17	0.49
	Assisted	0.07	0.12	0.49	0.96
	Observed	0.58	0.34	0.09	2.20
Airway intubation	Performed with or without supervision	0.08	0.00	0.13	0.00
	Assisted	0.04	0.08	0.31	0.48
	Observed	0.72	0.36	0.004*	4.86
Suturing lacerations	Performed with or without supervision	0.40	0.54	0.23	0.69
	Assisted	0.04	0.00	0.26	96.21
	Observed	0.36	0.33	0.42	1.13
Suturing operative wound closure	Performed with or without supervision	0.24	0.25	0.47	0.96
	Assisted	0.19	0.08	0.14	2.74
	Observed	0.42	0.36	0.33	1.30
Suturing OR drain	Performed with or without supervision	0.00	0.04	0.25	0.00
	Assisted	0.13	0.13	0.50	1.0
	Observed	0.54	0.29	0.05*	2.87
Hand tying	Performed with or without supervision	0.25	0.21	0.40	1.20
	Assisted	0.00	0.00	0.00	0.00
	Observed	0.48	0.40	0.29	1.39
Chest tube insertion	Performed with or without supervision	0.00	0.00	0.00	0.00
	Assisted	0.13	0.00	0.34	1.57
	Observed	0.33	0.21	0.18	1.9
Foley catheterization	Performed with or without supervision	0.20	0.32	0.22	0.60
	Assisted	0.04	0.04	0.50	0.92
	Observed	0.48	0.29	0.17	1.85

*p* < 0.05 is statistically significant

**Table 3:** Important procedures to include in future PDT

Procedure	Participants who rated procedural skills as important for PDT (% of participants) N = 26	Participants who rated procedural skills as very important for PDT (% of participants) N = 26	Total (% of participants) N = 26	Importance in PDT (mean response from 5 point Likert scale)
Intravenous line	23%	41%	64%	4.2
Suturing lacerations	39%	31%	70%	4.1
Assisting in surgery	39%	26%	65%	3.9
Post-operative wound care	38%	25%	63%	3.9
Foley catheterization	22%	26%	48%	3.8
Airway intubation	14%	23%	37%	3.7
Suturing operative wound closure	23%	23%	46%	3.6
Knowledge of surgical instruments	13%	18%	31%	3.4
Suturing a drain or tube to skin	14%	14%	28%	3.3
Hand tying	18%	17%	35%	3.3
Chest tube insertion	4%	13%	17%	3.0

**Table 4:** Ratings of importance for future PDT (mean rating on 5-point Likert scale)

Procedural skill/knowledge	Non-surgical elective students (N=7)	Surgical elective students (N=19)	*p*-value
Inserting IV lines	4.1	3.8	0.49
Airway intubation	3.9	3.1	0.15
Suturing lacerations	4.0	3.8	0.64
Surgical wound closure	3.9	3.3	0.38
Suturing drain or tube in OR	2.6	3.7	0.03*
Surgical assisting	4.0	3.6	0.48
Knowledge of surgical instruments	3.7	2.9	0.15
Hand tying	3.3	2.9	0.34
Chest tube insertion	3.6	2.3	0.025*
Foley catheterization	3.7	3.6	0.83
Post-operative wound care	3.9	3.6	0.71

* *p* < 0.05 is statistically significant

## Appendix A. Pre-Departure Procedural Skills Training for International Medical electives: A Survey of Canadian Medical Students

You are being invited to participate in a survey assessing procedural skills training prior to international electives for medical students. The purpose of this study is for curriculum development in pre-departure training. The Canadian Federation of Medical Students (CFMS) has issued guidelines on pre-departure training ( www.cfms.org ), however at no point do the guidelines mention actual procedural skills as an objective. This survey is designed to assess whether it is valuable to include procedures as part of future pre-departure training.

Please circle your answer.

1) What is your expected date of graduation from medical school?
  2013/2014/2015/2016
2) In what year of medical school did you undertake an international elective?
  ○ First year
  ○ Second year
  ○ Third year
  ○ Fourth year
3) What is your Gender? Female/male
4) What is your Age? 18–24/25–30/>30
5) What was the specialty of your international elective?
  ○ Surgery
  ○ Internal Medicine
  ○ Pediatrics
  ○ Family medicine
  ○ Other __________________________
6) Where did you go for your international elective?
  ○ South America/Central America/Caribbean
  ○ Middle East
  ○ Africa
  ○ Asia
  ○ Europe
  ○ Other (ex. USA?)
7) Please specify country ___________
7a. How long was the elective?
  2 weeks/3–4 wks/5–8 wks/>8wk
7b. Was your primary elective site at a:
  Community hospital or clinic/Medical school teaching hospital
8a. Does your medical school offer Pre-Departure Training before international medical electives? Yes/No
8b. If you answered Yes, does the Pre-Departure Training include procedural skills? Yes/No
9) Please specify the amount of time spent doing organized pre-departure procedural skills training at your medical school
  ○ None
  ○ Less than an hour
  ○ 1–4 hour workshop
  ○ 5–8 hours
  ○ > 8 hours
  ○ other_________________
10) Please indicate your level of exposure to various medical procedures PRIOR TO departing on your international elective
  **Table:**
  	Never Observed	Observed	Assisted	Performed under supervision	Performed without supervision
  Inserting IV Lines	1	2	3	4	5
  Airway Intubation	1	2	3	4	5
  Suturing lacerations	1	2	3	4	5
  Surgical wound closure	1	2	3	4	5
  Suturing a drain, chest tube or central line into position	1	2	3	4	5
  Hand tying knots	1	2	3	4	5
  Chest tube insertion	1	2	3	4	5
  Foley catheter insertion	1	2	3	4	5
11) Please indicate your level of exposure to various medical procedures DURING your international elective
  **Table:**
  	Never Observed	Observed	Assisted	Performed under supervision	Performed without supervision
  Inserting IV Lines	1	2	3	4	5
  Airway Intubation	1	2	3	4	5
  Suturing lacerations	1	2	3	4	5
  Surgical wound closure	1	2	3	4	5
  Suturing a drain, chest tube or central line into position	1	2	3	4	5
  Hand tying knots	1	2	3	4	5
  Chest tube insertion	1	2	3	4	5
  Foley catheter insertion	1	2	3	4	5
12) Please rate the level of importance for Canadian medical students to have pre-departure training in these procedural skills.
  **Table:**
  	Not important		Optional		Mandatory
  Inserting IV Lines	1	2	3	4	5
  Airway Intubation	1	2	3	4	5
  Suturing lacerations	1	2	3	4	5
  Surgical wound closure	1	2	3	4	5
  Suturing a drain, chest tube or central line into position	1	2	3	4	5
  Assisting in surgery (eg., retracting, “following” a suture, taking off clamps	1	2	3	4	5
  Knowledge of surgical instruments	1	2	3	4	5
  Hand tying	1	2	3	4	5
  Chest tube insertion	1	2	3	4	5
  Foley catheter insertion	1	2	3	4	5
  Post-operative wound care management	1	2	3	4	5
13) What proportion of your elective was spent in the operating room?
  ○ 0%
  ○ 0–10%
  ○ 10–20%
  ○ 20–30%
  ○ over 30% of the time
14) What percentage of the time did you perform medical procedures under appropriate supervision?
  ○ 0–10%
  ○ 10–20%
  ○ 20–50%
  ○ 50–80%
  ○ 80–100%
